# Analysis of the mediating effect of occupational burnout on the relationship between emotional labor and turnover intention among obstetric nurses

**DOI:** 10.3389/fpubh.2025.1631669

**Published:** 2025-09-19

**Authors:** Xueling Lv, Zhifang Ding, Tingting Wang, Meixue Zhang

**Affiliations:** ^1^Obstetrics Intensive Care Unit (OICU), North Department of Intensive Care Medicine, Fuyang People's Hospital, Fuyang, Anhui, China; ^2^Department of Obstetrics, Fuyang People's Hospital, Fuyang, Anhui, China

**Keywords:** emotional labor, occupational burnout, turnover intention, obstetric nurses, mediation analysis

## Abstract

**Objective:**

This study aimed to examine the mediating role of occupational burnout in the relationship between emotional labor and turnover intention among obstetric nurses in China.

**Methods:**

A cross-sectional survey was conducted among 205 obstetric nurses from six tertiary hospitals in Fuyang City, China. Validated scales were used to assess emotional labor (Emotional Labor Scale, ELS), occupational burnout (Maslach Burnout Inventory, MBI), and turnover intention (Turnover Intention Scale, TIS). Data were analyzed using Pearson correlation, univariate analysis, multiple linear regression, and structural equation modeling (SEM) with Bootstrap mediation analysis.

**Results:**

Significant associations were found between nurses' emotional labor and occupational burnout (*r* = 0.292, *P* < 0.01), occupational burnout and turnover intention (*r* = 0.152, *P* < 0.01), and emotional labor and turnover intention (*r* = 0.291, *P* < 0.01). The SEM demonstrated good fit indices (χ^2^/df = 2.032, RMSEA = 0.071, CFI = 0.957). Occupational burnout substantially mediated the relationship, accounting for 54.2% of the total effect of emotional labor on turnover intention (indirect effect: 0.110, 95% CI [0.069, 0.169], *P* < 0.001). Additionally, monthly night shifts (β = 0.35, *P* < 0.001) and professional title (β = 0.28, *P* < 0.01) emerged as significant predictors of turnover intention.

**Conclusion:**

Occupational burnout serves as a critical mediator between emotional labor and turnover intention among obstetric nurses. These findings highlight the urgent need for healthcare organizations to implement targeted interventions, including workload optimization, adaptive emotional labor training, and comprehensive psychological support programs. Such strategies are essential for reducing nurse turnover, ensuring adequate staffing levels, and ultimately improving maternal and neonatal care quality in healthcare systems.

## 1 Introduction

Obstetric nurses play a critical role in maternal and neonatal care, bearing high-intensity emotional labor and occupational stress. With the relaxation of childbirth policies, the workload of obstetric nurses has further increased, leading to a rising prevalence of occupational burnout and turnover intention (1, 2). In China's cultural context, despite policy shifts from the one-child policy to the three-child policy, fertility rates have continued to decline from 1.70 in 2017 to 1.07 in 2022, creating complex healthcare demands and increased pressure on obstetric nursing staff (3). Additionally, traditional Chinese cultural expectations for comprehensive maternal care and family-centered birthing experiences place additional emotional labor burdens on obstetric nurses, particularly in managing intergenerational family dynamics during childbirth (4). Occupational burnout is a common psychological health issue, particularly prominent among nursing professionals with high emotional labor demands (5–9). Studies have shown that obstetric nurses, when dealing with high-risk pregnancies, childbirth complications, and neonatal care, often experience significant psychological stress, which may trigger occupational burnout (10). Furthermore, frequent emotional labor requirements, such as displaying positive emotions while interacting with pregnant women and their families, may result in emotional exhaustion, exacerbating both occupational burnout and turnover intention (11).

The concept of emotional labor was first introduced by Hochschild (12), referring to the process by which individuals regulate their emotions in the workplace to display externally observable expressions and behaviors. Nurses‘ emotional labor typically involves providing emotional support to patients, such as conveying friendliness and empathy through smiling, eye contact, and verbal communication to alleviate patients' negative emotions and build trust (13). However, prolonged emotional labor may lead to emotional exhaustion and occupational burnout, particularly when emotional expressions do not align with genuine feelings (14). Studies have indicated that different emotional labor strategies—such as surface acting, deep acting, and genuine emotional expression—have distinct effects on occupational burnout and turnover intention (15). For instance, deep acting and genuine emotional expression can alleviate occupational burnout, whereas surface acting may exacerbate emotional exhaustion and increase turnover intention (16).

The theoretical foundation for examining burnout as a mediator between emotional labor and turnover intention is grounded in the Conservation of Resources (COR) theory and the Job Demands-Resources (JD-R) model (17, 18). According to COR theory, individuals strive to obtain, retain, and protect resources, and psychological stress occurs when resources are threatened or inadequate to meet demands (17). In nursing practice, emotional labor represents significant resource expenditure as nurses continuously invest emotional energy to manage their feelings while caring for patients. The JD-R model explains that job demands such as emotional labor requirements can lead to burnout when they exceed available resources, creating an energy depletion process (18). This resource depletion manifests as emotional exhaustion—the core component of burnout—which subsequently triggers a self-protective mechanism where nurses develop withdrawal intentions to preserve remaining resources (19). The mediation hypothesis suggests that workplace stressors (emotional labor) lead to psychological strain (burnout), which in turn results in behavioral outcomes (turnover intention), providing a logical explanation for why burnout serves as a critical pathway through which emotional labor influences nurses' decisions to leave their positions.

Professional Burnout, also known as job burnout, work exhaustion, or occupational burnout, was first proposed by American clinical psychologist Freudenberger in the 1970s. Maslach and Jackson (20) defined occupational burnout as a psychological syndrome comprising three dimensions: emotional exhaustion, depersonalization, and reduced personal accomplishment. Among these, emotional exhaustion is the core dimension of occupational burnout, referring to the depletion of emotional resources due to excessive emotional involvement. Studies have reported a high prevalence of occupational burnout among obstetric nurses, particularly under high workload conditions and frequent night shifts (1). Occupational burnout not only affects nurses' job satisfaction but is also significantly positively correlated with turnover intention (21). Therefore, investigating the mediating effect of occupational burnout in the relationship between emotional labor and turnover intention is crucial for understanding the turnover behavior of obstetric nurses.

Although previous studies have explored the impact of emotional labor on occupational burnout, few have systematically examined the mediating role of occupational burnout in the relationship between emotional labor and turnover intention, particularly among obstetric nurses. Based on the theoretical framework outlined above, this study proposes the following research hypotheses: (H1) Emotional labor significantly predicts turnover intention among obstetric nurses; (H2) Emotional labor significantly predicts occupational burnout among obstetric nurses; (H3) Occupational burnout significantly predicts turnover intention among obstetric nurses; (H4) Occupational burnout mediates the relationship between emotional labor and turnover intention; and (H5) Different emotional labor strategies (surface acting, deep acting, and genuine emotional expression) exert distinct mediating effects through burnout on turnover intention, with surface acting showing stronger positive mediation compared to deep acting and genuine emotional expression. This study aims to analyze the relationships among emotional labor, occupational burnout, and turnover intention among obstetric nurses using a cross-sectional design and to verify these hypothesized mediating effects. The findings will provide a basis for emotional management and burnout intervention strategies, contributing to improving the work environment of obstetric nurses, reducing turnover intention, and enhancing job satisfaction.

## 2 Subjects and methods

### 2.1 Study subjects

This study employed a cross-sectional descriptive design using convenience sampling methodology. The study was conducted across six tertiary hospitals in Fuyang City, Anhui Province, China, between November 2024 and January 2025. A total of 205 obstetric nurses were recruited as study participants from the selected healthcare facilities.

#### 2.1.1 Inclusion criteria

(1) Possession of a nurse practitioner qualification certificate; (2) Continuous engagement in obstetric nursing for ≥1 year with regular shift participation; (3) Informed consent, and voluntary participation in the study.

#### 2.1.2 Exclusion criteria

(1) Nurses unable to work normally due to maternity leave, sick leave, or other reasons; (2) Nurses who experienced significant stress events (e.g., bereavement, major illness) in the past 6 months; (3) Nurses undergoing further training, rotations, or internships.

The sample size was calculated using G^*^Power 3.1.9.7 software based on multiple regression analysis requirements. For detecting a medium effect size (*f*^2^ = 0.15) with α = 0.05, power = 0.80, and considering the number of predictors in our regression models, the minimum required sample size was determined to be 68 participants (22). Additionally, following the recommendation by Tabachnick and Fidell (23) for multiple regression analysis, a minimum of *N* ≥ 50 + 8m (where m is the number of independent variables) is required. With up to nine demographic and scale variables as predictors, this yields a minimum sample size of 122 participants. Considering a potential 15% attrition rate common in cross-sectional survey studies, the target sample size was set at 140 participants. Our final sample of 205 participants substantially exceeds both requirements, providing statistical power >0.95 and enhanced protection against model instability due to random data fluctuations. Based on practical feasibility, a total of 205 valid samples were included, meeting statistical requirements. This study was approved by the Ethics Committee of Fuyang People's Hospital (Ethical Approval No.: [2024]192), with all participants providing informed consent. Data were anonymized and strictly kept confidential.

### 2.2 Survey methods

#### 2.2.1 Survey instruments

##### 2.2.1.1 *Demographic characteristics survey*

This investigator-developed instrument encompassed key sociodemographic variables: gender, age, professional title, level, way of holding office, marital status, education level, working years, and number of night shifts per month.

##### 2.2.1.2 *Emotional Work Assessment Tool*

The investigation employed the Emotional Labor Scale (ELS) by Gosserand and Diefendorff (24), culturally adapted for Chinese populations by Bai (25). This 14-item instrument measures three dimensions: surface acting, deep acting, and genuine emotional expression, using a 5-point Likert scale (Cronbach's α = 0.853). Detailed scoring procedures are provided in Supplementary material.

##### 2.2.1.3 *Occupational exhaustion measurement*

Burnout assessment utilized the Maslach Burnout Inventory (MBI) (26), adapted by Feng et al. (27). This 22-item tool evaluates emotional exhaustion, depersonalization, and personal accomplishment using a 7-point frequency scale. Burnout severity classification and diagnostic thresholds are detailed in Supplementary material (28). The MBI demonstrated good reliability with Cronbach's α coefficients of 0.861 (overall scale) and 0.823 (emotional exhaustion subscale).

##### 2.2.1.4 *Turnover Intention Assessment*

The Turnover Intention Scale (TIS) (29), adapted by Li et al. (30), was employed to assess three domains: position retention probability, career transition impetus, and external opportunity acquisition feasibility. The 6-item instrument uses a 4-point frequency scale (Cronbach's α = 0.773). Complete scoring details are available in Supplementary material.

#### 2.2.2 Data collection methods

After obtaining approval from the nursing department of six tertiary hospitals in Fuyang City, this study distributed electronic questionnaires via the Wenjuanxing platform. A standardized set of instructions was provided, and hospital representatives shared the QR code of the questionnaire with eligible obstetric nurses. Participants completed the questionnaire anonymously and could consult the hospital representatives for any questions. Collected data were reviewed by researchers. All of the collected questionnaires were valid.

### 2.3 Statistical methods

Data analysis was performed using SPSS 27.0. Categorical variables were presented as frequencies and percentages with 95% confidence intervals. Normally distributed continuous variables were expressed as mean ± SD, while non-normally distributed variables were reported as median (IQR). Prior to parametric testing, assumptions of normality (Shapiro-Wilk test), homoscedasticity (Levene's test), and multicollinearity (variance inflation factor <10) were evaluated and confirmed. Common method bias was assessed using Harman's single-factor test to address potential inflation of correlations due to single-source, cross-sectional data collection. Relationships between emotional labor, job burnout, and turnover intention were assessed using Pearson's correlation analysis. Group comparisons were conducted using independent *t*-tests (for two groups) or one-way ANOVA (for three or more groups) with SNK *post-hoc* tests, assuming normality and homogeneity of variance. Effect sizes were reported using Cohen's d for *t*-tests and eta-squared (η^2^) for ANOVA analyses. For multivariate linear regression, variable selection was based on: (1) theoretical relevance from existing literature demonstrating associations with turnover intention, and (2) statistical significance (*P* < 0.05) in univariate analyses. Specifically, demographic variables showing significant univariate associations (professional title, hierarchical level, monthly night shifts) were included alongside theoretically important constructs (emotional labor total score, job burnout total score). Age was excluded from the final model due to multicollinearity concerns (VIF > 5) with hierarchical level and work experience. Data were examined for outliers using standardized residuals (±3 SD criterion). Regression results are presented with standardized coefficients, 95% confidence intervals, and significance levels. To examine the mediating role of job burnout, structural equation modeling (SEM) was implemented in AMOS 24.0. Bootstrap analysis with 5,000 resamples was used to test mediation effects, with 95% confidence intervals (bias-corrected). Statistical significance was set at *P* < 0.05 (two-tailed).

## 3 Results

### 3.1 Descriptive analysis of basic information

The study included 205 obstetric nurses, with baseline characteristics presented in Table 1. Regarding age distribution, the most common age group was 31–40 years (*n* = 111, 54.1%), followed by 21–30 years (*n* = 68, 33.2%). The most frequent professional title was nurse-in-charge (*n* = 109, 53.2%), while junior nurses comprised 32.7% (*n* = 67) of the sample. In terms of nursing hierarchical levels, N4 represented the largest group (*n* = 73, 35.6%), followed by N3 (*n* = 64, 31.2%). The majority of participants were contract employees (*n* = 168, 82.0%) compared to permanent staff (*n* = 37, 18.0%). Most nurses were married (*n* = 158, 77.1%), with single nurses accounting for 20.5% (*n* = 42). Educational attainment was predominantly bachelor's degree or higher (*n* = 166, 81.0%), while associate degree holders comprised 17.5% (*n* = 36). The most common work experience category was 6–10 years (*n* = 86, 42.0%), followed by 11–15 years (*n* = 45, 22.0%). Regarding monthly night shift frequency, the highest proportion worked 8–12 shifts (*n* = 74, 36.1%), followed by fewer than eight shifts (*n* = 67, 32.7%), while 16.6% (*n* = 34) worked no night shifts.

**Table 1:** Descriptive statistics of participants and study variables.

**Characteristic**	***n* (%) or Mean ±SD**	**Mean item score**
**Demographics**		
Age (years)		–
21–30	68 (33.2)	
31–40	111 (54.1)	
41–50	22 (10.7)	
51–60	4 (2.0)	
**Professional title**		
Nurse	18 (8.7)	
Junior nurse	67 (32.7)	
Nurse-in-charge	109 (53.2)	
Deputy chief nurse and above	11 (5.4)	
**Nursing hierarchical level**		
N1	20 (9.8)	
N2	38 (18.5)	
N3	64 (31.2)	
N4	73 (35.6)	
N5	10 (4.9)	
**Employment type**		
Permanent	37 (18.0)	
Contract	168 (82.0)	
**Marital status**		
Single	42 (20.5)	
Married	158 (77.1)	
Divorced	5 (2.4)	
**Education level**		
Technical school	3 (1.5)	
Associate degree	36 (17.5)	
Bachelor's degree and above	166 (81.0)	
**Work experience (years)**		
1–5	35 (17.0)	
6–10	86 (42.0)	
11–15	45 (22.0)	
16–20	18 (8.8)	
>20	21 (10.2)	
**Night shifts per month**		
None	34 (16.6)	
<8	67 (32.7)	
8–12	74 (36.1)	
12–18	30 (14.6)	
**Study variables**		
Emotional labor (total score)	44.66 ± 11.70	3.19 ± 0.84
Surface acting	22.01 ± 6.30	3.14 ± 0.90
Deep acting	12.65 ± 3.66	3.16 ± 0.91
Genuine expression	10.00 ± 2.66	3.33 ± 0.89
Job burnout (total score)	52.91 ± 13.62	2.41 ± 0.62
Emotional exhaustion	18.17 ± 10.10	2.02 ± 1.12
Personal accomplishment	29.44 ± 10.66	3.68 ± 1.33
Depersonalization	5.30 ± 4.09	1.06 ± 0.82
Turnover intention	13.00 ± 3.65	2.17 ± 0.61
**Burnout level**		**95% CI**
No burnout	54 (26.3)	20.4–32.9
Mild burnout	73 (35.6)	29.0–42.7
Moderate burnout	52 (25.4)	19.6–31.9
Severe burnout	26 (12.7)	8.5–18.1
Total burnout	151 (73.7)	67.1–79.6

### 3.2 Scores of emotional labor, job burnout, and turnover intention

Table 1 presents the descriptive statistics for all study variables. The mean emotional labor total score was 44.66 ± 11.70, with a mean item score of 3.19 ± 0.84. Among emotional labor subdimensions, surface acting demonstrated the highest score (22.01 ± 6.30, mean item score 3.14 ± 0.90), followed by deep acting (12.65 ± 3.66, mean item score 3.16 ± 0.91) and genuine expression (10.00 ± 2.66, mean item score 3.33 ± 0.89). The overall job burnout score was 52.91 ± 13.62 with a mean item score of 2.41 ± 0.62. Within job burnout dimensions, personal accomplishment showed the highest score (29.44 ± 10.66, mean item score 3.68 ± 1.33), followed by emotional exhaustion (18.17 ± 10.10, mean item score 2.02 ± 1.12), and depersonalization (5.30 ± 4.09, mean item score 1.06 ± 0.82). The turnover intention score was 13.00 ± 3.65 with a mean item score of 2.17 ± 0.61. Notably, 106 participants (51.7%) demonstrated turnover intention scores above the scale midpoint, indicating moderate to high levels of turnover intention.

According to Table 1, 54 participants (26.3%) showed no job burnout, whereas 151 participants (73.7%) exhibited varying degrees of job burnout. Specifically, mild burnout was observed in 73 participants (35.6%), moderate burnout in 52 participants (25.4%), and severe burnout in 26 participants (12.7%).

### 3.3 Correlation analysis

Table 2 presents the correlation analysis results between emotional labor, job burnout, and turnover intention. Significant correlations were found among the variables. All dimensions and total scores of emotional labor were positively correlated with job burnout total score and its subdimensions (emotional exhaustion and depersonalization), with correlation coefficients ranging from *r* = 0.129 to *r* = 0.315 (*P* < 0.05 or *P* < 0.01). Job burnout subdimensions showed differential associations with turnover intention: emotional exhaustion (*r* = 0.372, *P* < 0.01) and depersonalization (*r* = 0.381, *P* < 0.01) were positively correlated with turnover intention, while personal accomplishment was negatively correlated with turnover intention (*r* = −0.244, *P* < 0.01). Among emotional labor dimensions, deep acting exhibited the strongest correlation with turnover intention (*r* = 0.330, *P* < 0.01), followed by surface acting (*r* = 0.285, *P* < 0.01) and the emotional labor total score (*r* = 0.291, *P* < 0.01). Notably, deep acting demonstrated the strongest correlation with turnover intention, suggesting this emotional labor strategy may be particularly problematic for nurse retention.

**Table 2:** Correlation analysis of emotional labor, job burnout, and turnover intention.

**Variable**	**1**	**2**	**3**	**4**	**5**	**6**	**7**	**8**	**9**
1. Emotional labor	1.000								
2. Surface acting	0.840^**^	1.000							
3. Deep acting	0.733^**^	0.596^**^	1.000						
4. Genuine expression	0.561^**^	0.442^**^	0.429^**^	1.000					
5. Job burnout	0.292^**^	0.272^**^	0.278^**^	0.210^**^	1.000				
6. Emotional exhaustion	0.231^**^	0.221^**^	0.296^**^	0.129^*^	0.387^**^	1.000			
7. Personal accomplishment	0.056	0.041	−0.015	0.103^*^	0.327^**^	−0.290^**^	1.000		
8. Depersonalization	0.249^**^	0.242^**^	0.315^**^	0.120^*^	0.305^**^	0.560^**^	−0.308^**^	1.000	
9. Turnover intention	0.291^**^	0.285^**^	0.330^**^	0.162^**^	0.152^**^	0.372^**^	−0.244^**^	0.381^**^	1.000

^**^P < 0.01, ^*^P < 0.05.

### 3.4 Univariate analysis

Table 3 presents the univariate analysis results comparing emotional labor, job burnout, and turnover intention across demographic and work-related characteristics. The analysis was conducted using one-way ANOVA with effect sizes reported as *F*-statistics.

**Table 3:** Univariate analysis of emotional labor, job burnout, and turnover intention.

**Variable**	**Age**	**Professional title**	**Hierarchical level**	**Employment type**	**Marital status**	**Education level**	**Work experience**	**Night shifts**
**Emotional labor**								
*F*-statistic	3.023^*^	1.237	2.303	0.195	2.492	0.295	1.829	3.276^*^
η^2^	0.025	0.011	0.020	0.002	0.021	0.003	0.016	0.027
**Job burnout**								
*F*-statistic	1.610	2.203	0.363	0.666	2.731	0.002	0.791	3.096^*^
η^2^	0.014	0.019	0.003	0.006	0.023	0.000	0.007	0.026
**Turnover intention**								
*F*-statistic	1.361	8.526^***^	3.800^***^	1.571	0.536	1.054	1.322	8.304^***^
η^2^	0.012	0.071	0.032	0.014	0.005	0.009	0.012	0.069

^***^P < 0.001, ^*^P < 0.05. η^2^ = eta-squared (effect size); 0.01 = small effect, 0.06 = medium effect, 0.14 = large effect.

Emotional labor differences: Significant variations in emotional labor scores were observed across age groups (*F* = 3.023, *P* < 0.05, η^2^ = 0.025) and monthly night shift frequency (*F* = 3.276, *P* < 0.05, η^2^ = 0.027). *Post-hoc* Tukey tests revealed that nurses aged 41–50 years reported significantly higher emotional labor scores compared to those aged 20–30 years (*P* < 0.05). Regarding night shifts, nurses working more than eight night shifts per month demonstrated significantly higher emotional labor scores than those working 0–8 shifts (*P* < 0.05).

#### 3.4.1 Job burnout differences

Monthly night shift frequency was the only variable showing significant association with job burnout scores (*F* = 3.096, *P* < 0.05, η^2^ = 0.026). *Post-hoc* analysis indicated that nurses working more than 8 night shifts per month experienced significantly higher burnout levels compared to those working fewer night shifts (*P* < 0.05).

#### 3.4.2 Turnover intention differences

Three variables demonstrated significant effects on turnover intention. Professional title showed the strongest association (*F* = 8.526, *P* < 0.001, η^2^ = 0.071), with *post-hoc* tests revealing that staff nurses reported significantly higher turnover intention than charge nurses and nurse managers (*P* < 0.001). Hierarchical level also significantly influenced turnover intention (*F* = 3.800, *P* < 0.001, η^2^ = 0.032), where lower-level positions showed higher turnover intention. Monthly night shift frequency demonstrated a substantial effect (*F* = 8.304, *P* < 0.001, η^2^ = 0.069), with nurses working more frequent night shifts expressing greater turnover intention.

Employment type, marital status, education level, and work experience showed no significant associations with any of the outcome variables (all *P* > 0.05).

### 3.5 Multiple linear regression analysis

To further explore the independent effects of variables on turnover intention, multiple linear regression analysis was conducted with turnover intention as the dependent variable (Table 4). Prior to model construction, multicollinearity diagnostics were performed using VIF analysis. Age was excluded from the final model due to high multicollinearity with work experience (VIF = 4.82), exceeding the recommended threshold of 4.0. The final model included significant predictors from univariate analysis (professional title, hierarchical level, monthly night shifts) and key theoretical variables (emotional labor, job burnout) as independent variables, with all remaining VIF values below 2.5, indicating acceptable multicollinearity levels.

**Table 4:** Multiple linear regression analysis of turnover intention.

**Independent variable**	**β-coefficient**	**SE**	***t*-value**	***P*-value**	**95% CI**	**VIF**
Professional title	0.28	0.09	3.11	0.002	[0.10, 0.46]	1.85
Hierarchical level	0.15	0.08	1.88	0.061	[−0.01, 0.31]	2.12
Monthly night shifts	0.35	0.07	5.00	<0.001	[0.21, 0.49]	1.67
Emotional labor	0.18	0.09	1.96	0.052	[−0.01, 0.37]	2.34
Job burnout	0.22	0.10	2.20	0.029	[0.02, 0.42]	1.92
Constant	1.05	0.45	2.33	0.021	[0.16, 1.94]	–

Model fit indices: *R*^2^ = 0.42, adjusted *R*^2^ = 0.39, F = 12.56, *P* < 0.001. VIF, Variance Inflation Factor; all VIF values <2.5 indicate acceptable multicollinearity levels.

Results indicated that professional title (β = 0.28, 95% CI [0.10, 0.46], *P* < 0.01) and monthly night shifts (β = 0.35, 95% CI [0.21, 0.49], *P* < 0.001) were significant predictors of turnover intention, with monthly night shifts demonstrating the strongest predictive effect. Job burnout showed a significant positive association (β = 0.22, 95% CI [0.02, 0.42], *P* < 0.05), while hierarchical level approached significance (β = 0.15, 95% CI [-0.01, 0.31], *P* = 0.061). Emotional labor did not reach statistical significance (β = 0.18, 95% CI [−0.01, 0.37], *P* = 0.052), though the confidence interval suggests a potential positive association. The overall model was statistically significant and explained 42% of the variance in turnover intention (*R*^2^ = 0.42, adjusted *R*^2^ = 0.39, *F* = 12.56, *P* < 0.001).

### 3.6 Mediation effect analysis

**Figure 1:**
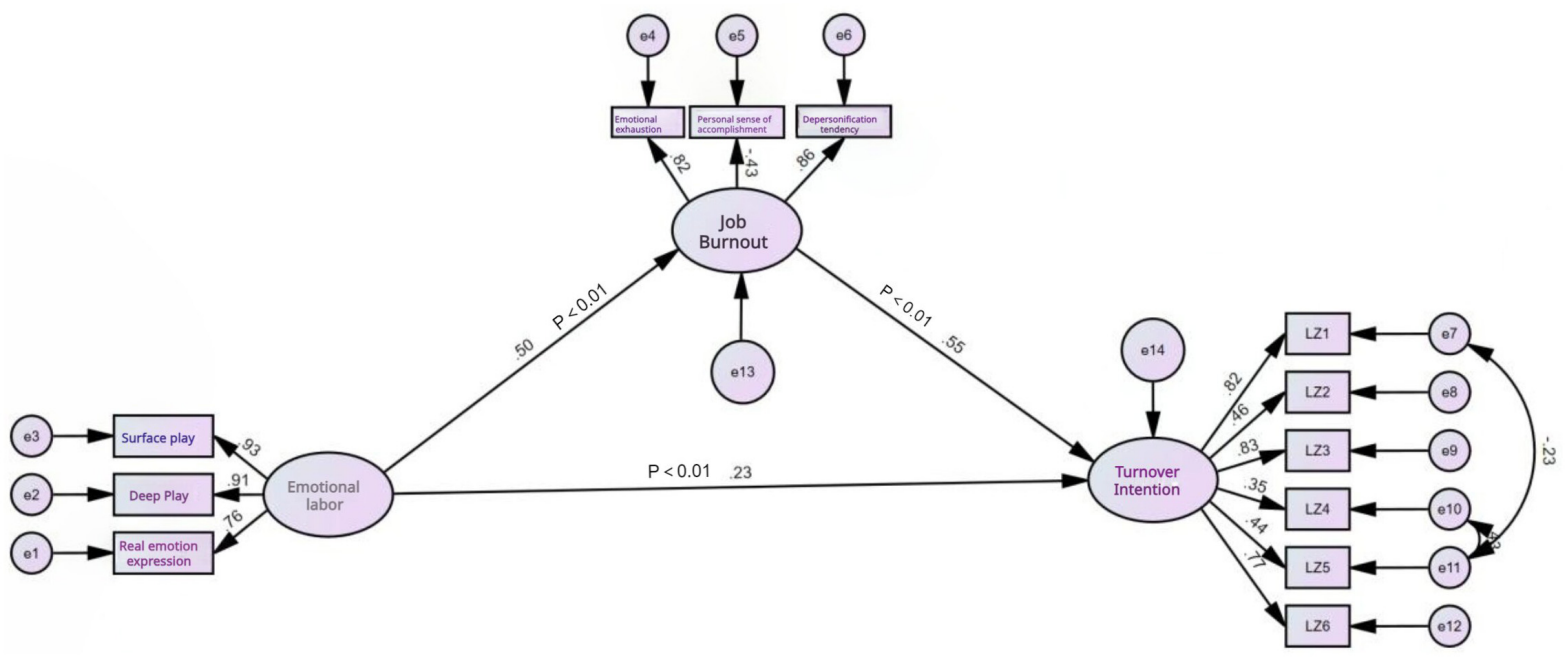
Mediating effect of occupational burnout between emotional labor and turnover intention. The figure illustrates the structural equation model (SEM) analyzing the relationships between emotional labor, occupational burnout, and turnover intention. Emotional labor is measured through its dimensions: surface acting, deep acting, and genuine emotional expression, which are depicted as latent variables. Occupational burnout includes emotional exhaustion, depersonalization, and personal accomplishment, while turnover intention is represented as a separate latent variable. Standardized path coefficients between the variables are shown.

To further explore the interrelationships among emotional labor, job burnout, and turnover intention, structural equation modeling was employed using AMOS 24.0. The model demonstrated good fit indices: χ^2^/df = 2.032, GFI = 0.930, CFI = 0.957, TLI = 0.942, and RMSEA = 0.071, all meeting acceptable criteria. The standardized path coefficients were 0.23 (emotional labor to job burnout), 0.50 (job burnout to turnover intention), and 0.55 (emotional labor to turnover intention), with all paths significant at *P* < 0.05 (Figure 1).

The mediation analysis revealed that job burnout significantly mediated the relationship between emotional labor and turnover intention (Table 5). The indirect effect was 0.110 (SE = 0.025, 95% CI [0.069, 0.169], *P* < 0.001), accounting for 54.2% of the total effect, while the direct effect remained significant (0.093, SE = 0.032, 95% CI [0.033, 0.162], *P* = 0.004), representing 45.8% of the total effect. The total effect was 0.203 (SE = 0.031, 95% CI [0.152, 0.276], *P* < 0.001). Since the confidence intervals for both direct and indirect effects exclude zero, this confirms partial mediation, indicating that job burnout serves as a significant mediator while emotional labor also maintains a direct influence on turnover intention.

**Table 5:** Mediation effect test.

**Effect type**	**Point estimate**	**SE**	**95% CI**	***P*-value**	**Effect proportion**
Indirect effect	0.110	0.025	(0.069, 0.169)	<0.001	54.2%
Direct effect	0.093	0.032	(0.033, 0.162)	0.004	45.8%
Total effect	0.203	0.031	(0.152, 0.276)	<0.001	100.0%

### 3.7 Mediation analysis: job burnout as mediator

To examine the differential pathways of emotional labor strategies on turnover intention, mediation analysis was conducted using the PROCESS macro. Results revealed that job burnout significantly mediated the relationship between deep acting and turnover intention (indirect effect = 0.087, 95% CI [0.032, 0.156]), with the mediation effect being stronger than for surface acting (indirect effect = 0.061, 95% CI [0.018, 0.118]) and genuine expression (indirect effect = 0.042, 95% CI [0.008, 0.089]). Deep acting showed both the strongest direct effect (β = 0.247, *P* < 0.01) and indirect effect through job burnout on turnover intention.

## 4 Discussion

This study reveals the complex relationship between emotional labor, job burnout, and turnover intention, further validating the partial mediating role of job burnout between emotional labor and turnover intention. The results show that 106 participants (51.7%) had an average score of>2 for their intention to resign, indicating that obstetric nurses have a high intention to resign. This can be analyzed from the perspective of reducing emotional labor and alleviating job burnout to reduce their intention to resign. The total score of emotional labor and its sub-dimensions are significantly positively correlated with job burnout, while the intensification of job burnout directly increases the turnover intention of obstetric nurses. This finding is consistent with the conclusions of Kashif and Foong (1) and Opoku et al. (2), indicating that obstetric nurses, due to prolonged exposure to high emotional labor environments, are prone to developing turnover tendencies through the mediating pathway of job burnout. Notably, surface acting, as one of the core strategies of emotional labor, shows the most significant positive correlation with job burnout and turnover intention. This may be because surface acting requires nurses to continuously suppress their genuine emotions, leading to psychological resource depletion (31). Deep performance and genuine emotional expression also have a negative impact on job burnout. aligning with Zhang et al.'s (32) research. Surface acting, as one of the core strategies of emotional labor, shows the most significant positive correlation with job burnout and turnover intention” requires contextual analysis within our study results. Our findings revealed that deep acting actually demonstrated the strongest correlation with turnover intention, which differs from Zhang's (32) results. This discrepancy may be attributed to sample differences—our study specifically focused on obstetric nurses who face unique emotional demands including emergency deliveries and perinatal loss, whereas Zhang's study examined general nursing populations. The specialized nature of obstetric care may make deep acting more psychologically demanding due to the intensity of maternal care situations. Additionally, our study context within Chinese obstetric departments, characterized by high patient volumes and staffing shortages, may create conditions where the psychological resources required for deep acting become particularly depleted.

This study found that the overall incidence rate of job burnout reached 73.7%, which far exceeds rates reported among general healthcare workers. This elevated burnout rate may be attributed to structural issues within China's obstetric nursing workforce, including chronic understaffing and high patient-to-nurse ratios that intensify workload pressures (33). The shortage of qualified obstetric nurses has created unsustainable working conditions that contribute to emotional exhaustion and burnout (34). This aligns with Maslach's (20) theoretical framework, which posits that emotional exhaustion, as the core dimension of job burnout, indirectly exacerbates turnover tendencies by weakening nurses' work engagement and self-efficacy. Furthermore, the significant influence of the number of night shifts per month and professional title on turnover intention further corroborates Doleman et al.'s (35) conclusion: senior nurses, who bear greater responsibilities and face more complex work scenarios, may experience more pronounced job burnout and turnover intention. These results suggest that workload distribution and career development support should be key areas of intervention.

Our findings revealed that deep acting demonstrated the strongest correlation with turnover intention among the three emotional labor strategies, warranting theoretical explanation within the obstetric nursing context. From a theoretical perspective, deep acting requires nurses to genuinely modify their internal emotional states to align with organizational display rules, which demands substantial psychological resources according to the Conservation of Resources theory (17). Unlike surface acting, which involves only superficial emotional expression, deep acting creates more severe resource depletion because it requires authentic emotional investment while potentially conflicting with nurses' genuine feelings (36). In the obstetric context, this may be particularly problematic due to the unique emotional demands of maternal care. Obstetric nurses frequently encounter emotionally intense situations including emergency deliveries, fetal distress, and perinatal loss, where they must maintain composure and provide emotional support while managing their own emotional responses (37). The expectation to genuinely feel positive emotions during traumatic birth experiences or when supporting grieving families creates particularly intense emotional labor demands. The intimate nature of obstetric care, involving highly personal and vulnerable moments in families' lives, may intensify the pressure for authentic emotional engagement, making deep acting more psychologically demanding than in other healthcare settings (38). This emotional intensity, combined with the unpredictable nature of obstetric emergencies, may explain why deep acting shows the strongest association with burnout and turnover intention in this specialized nursing population.

The study also found that while the direct effect of emotional labor on turnover intention did not reach statistical significance, its indirect effect through the mediating pathway of job burnout accounted for 54.2% of the total effect. This result reveals the indirect mechanism by which emotional labor influences turnover intention, consistent with Martínez-Íñigo et al.'s (39) “emotional regulation resource depletion model.” When nurses adopt surface acting strategies over long periods, the psychological costs of emotional labor are likely to impact turnover decisions primarily through the mediating pathway of job burnout rather than through direct effects. This mechanism is particularly evident in obstetric care, where the nature of the work requires nurses to frequently manage the emotional fluctuations of pregnant and postpartum women, intensifying the demands of emotional labor (40). The non-significant direct effect of emotional labor on turnover intention (*p* = 0.052) indicates that emotional demands alone do not directly drive nurses to leave, but rather exert their influence primarily through the burnout pathway, thereby highlighting the essential mediating role of burnout in this relationship. This finding reinforces that emotional labor's impact on turnover operates through resource depletion and subsequent burnout rather than as a direct predictor, which strongly supports Martínez-Íñigo's (39) theoretical framework emphasizing the cascading effects of emotional regulation demands.

These findings suggest that job burnout serves as a critical pathway through which emotional labor influences turnover intention. Given that the indirect effect accounts for over half of the total relationship, interventions targeting burnout reduction—such as workload management, emotional support programs, and stress management training—could substantially mitigate turnover intention among nurses experiencing high emotional labor demands. This mediation pathway provides healthcare administrators with a clear intervention target: reducing job burnout may effectively break the link between unavoidable emotional labor and nurse turnover.

Based on the above findings, it is recommended that hospital administrators improve the occupational health of obstetric nurses through three approaches: First, optimize scheduling systems to reduce the frequency of night shifts for senior nurses and alleviate the pressure of emotional labor through flexible work arrangements; Second, contrary to our initial suggestion, recent intervention studies indicate that surface acting training combined with emotional intelligence programs may be more effective than promoting deep acting, as deep acting can paradoxically increase emotional exhaustion (41). These programs can be integrated into existing hospital protocols through monthly unit-based training sessions and incorporated into annual competency requirements. Third, establish specific psychological support interventions such as mindfulness-based stress reduction (MBSR) programs and Employee Assistance Programs (EAP), which have demonstrated effectiveness in reducing healthcare worker burnout (42). MBSR protocols can be implemented through 8-week structured programs during shift changes, while cognitive-behavioral approaches can be delivered through brief intervention modules during staff meetings. Regular burnout screening should be coupled with these evidence-based interventions rather than generic counseling approaches, with screening tools administered quarterly and linked to immediate access to targeted interventions. These targeted measures would help address both the structural and individual factors contributing to the high burnout rates observed in obstetric nursing.

## Limitations

Despite providing important evidence for understanding the turnover behavior of obstetric nurses, this study has certain limitations. First, the cross-sectional design limits the ability to infer causal relationships; future studies could use longitudinal research to track the dynamic changes in job burnout and its long-term impact on turnover intention. Second, the convenience sampling method introduces significant limitations to internal validity and generalizability. The non-random sampling approach creates potential selection bias, as participants were recruited from six tertiary hospitals in a single city within Anhui Province, which may not represent nurses working in different healthcare settings, geographic regions, or hospital levels (primary care facilities, secondary hospitals, or rural settings). Additionally, the voluntary participation design may have resulted in response bias, where nurses with stronger opinions about emotional labor, job satisfaction, or workplace conditions were more likely to participate, potentially skewing our findings. Third, the study did not include potential variables such as perceived organizational support or job satisfaction, which may partially obscure the complex association between emotional labor and turnover intention. Fourth, the convenience sampling limits the external validity of our findings, as the results may not be generalizable to the broader population of obstetric nurses beyond the specific institutional and regional context studied. Future research could employ probability sampling methods, expand the sample scope to include diverse healthcare settings and geographic regions, and integrate multidimensional variables to construct a more comprehensive theoretical model while enhancing both internal and external validity. Additionally, our study did not distinguish between job burnout and potential underlying mental health conditions such as depression and anxiety. The emotional exhaustion component of burnout shares conceptual overlap with depressive symptoms, and both depression and anxiety may independently influence emotional labor experiences and turnover intention. Future studies should include validated measures of mental health symptoms to differentiate between work-specific burnout and general psychological distress, allowing for a more precise understanding of the mechanisms linking emotional labor to turnover intention. While our mediation analysis controlled for key demographic variables, future studies with larger samples should examine potential moderated mediation effects, particularly how employment type, professional rank, and night shift frequency may influence the emotional labor-burnout-turnover pathway. Additionally, all data were collected through self-report questionnaires at a single time point, which may have inflated correlations between variables due to common method bias. While Harman's single-factor test indicated that common method variance was not a major concern in our study, the cross-sectional design limits our ability to establish causal relationships between emotional labor, burnout, and turnover intention. Future research should employ longitudinal designs or multi-source data collection methods to better address potential method bias and establish temporal precedence among study variables.

## Conclusions

In conclusion, this study deepens the understanding of the turnover mechanism of obstetric nurses by validating the mediating effect of job burnout. The findings provide empirical support for emotional labor theory and lay a scientific foundation for developing targeted intervention strategies. Future research could further explore how factors such as work environment optimization and organizational culture shaping influence turnover intention through the regulation of mediating pathways, thereby providing more comprehensive decision-making references for nursing human resource management.

## Data Availability

The original contributions presented in the study are included in the article/Supplementary material, further inquiries can be directed to the corresponding author.
