# Supplementary material for: Analysis of the mediating effect of occupational burnout on the relationship between emotional labor and turnover intention among obstetric nurses
Source: Front Public Health. 2025 Sep 19;13:1631669. doi: 10.3389/fpubh.2025.1631669 (PMC12491317; doi:10.3389/fpubh.2025.1631669)
Supplement: Supplementary file 1 [file Data_Sheet_1.docx]

**Supplementary Material**

**Emotional Labor Scale Scoring Details**

- Surface acting subscale: items 1-7 (score range: 7-35)
- Deep acting subscale: items 8-11 (score range: 8-20)
- Genuine emotional expression subscale: items 12-14 (score range: 3-15)
- Total score range: 14-70 points
- Scoring: 1=completely disagree to 5=completely agree
- Interpretation: Higher scores indicate greater emotional labor demands

**MBI Burnout Classification System**

Emotional exhaustion: 9 items (score range: 9-63)

Depersonalization: 5 items (score range: 5-35)

Personal accomplishment: 8 items (score range: 8-56, reverse scored)

Diagnostic thresholds (Ye et al. [19]):

- Emotional exhaustion ≥27
- Depersonalization ≥8
- Personal accomplishment ≤24

Severity classification:

- Asymptomatic: all domains below threshold
- Mild: one domain above threshold
- Moderate: two domains above threshold
- Severe: all three domains above threshold

Overall burnout incidence rate calculation:

Rate = (Mild + Moderate + Severe cases) / Total cases × 100%

**Turnover Intention Scale Scoring**

- Current position retention: items 1-2 (score range: 2-8)
- Career transition impetus: items 3-4 (score range: 2-8)
- External opportunity acquisition: items 5-6 (score range: 2-8)
- Total score range: 6-24 points
- Scoring: 1=never to 4=often
- Interpretation: Higher scores indicate greater turnover intention
